# Effects of soiling on photovoltaic (PV) modules in the Atacama Desert

**DOI:** 10.1038/s41598-018-32291-8

**Published:** 2018-09-17

**Authors:** R. R. Cordero, A. Damiani, D. Laroze, S. MacDonell, J. Jorquera, E. Sepúlveda, S. Feron, P. Llanillo, F. Labbe, J. Carrasco, J. Ferrer, G. Torres

**Affiliations:** 1 Universidad de Santiago de Chile, Av. Bernardo O’Higgins 3363, Santiago, Chile; 2 Center for Environmental Remote Sensing, Chiba University, Chiba, Japan; 3 Instituto de Alta Investigación, CEDENA, Universidad de Tarapacá, Casilla 7D, Arica, Chile; 4 Centro de Estudios Avanzados en Zonas Áridas (CEAZA), Raúl Bitrán 1305, La Serena, Chile; 5 Universidad Técnica Federico Santa María, Av. Espana 1680, Valparaíso, Chile; 6 Universidad de Magallanes, Av. Bulnes 1855, Punta Arenas, Chile; 7 Direccion Meteorologica de Chile, Av. Portales 3450, Santiago, Chile

## Abstract

Soiling by dry deposition affects the power output of photovoltaic (PV) modules, especially under dry and arid conditions that favor natural atmospheric aerosols (wind-blown dust). In this paper, we report on measurements of the soiling effect on the energy yield of grid-connected crystalline silicon PV modules deployed in five cities across a north-south transect of approximately 1300 km in the Atacama Desert ranging from latitude 18°S to latitude 30°S. Energy losses were assessed by comparing side-by-side outputs of four co-planar PV modules. Two of the PV modules of the array were kept clean as a control, while we allowed the other two to naturally accumulate soiling for 12 months (from January 2017 to January 2018). We found that the combination of high deposition rates and infrequent rainfalls led to annual energy losses that peaked at 39% in the northern coastal part of the desert. In contrast, annual energy losses of 3% or less were measured at relatively high-altitude sites and also at locations in the southern part of the desert. For comparison, soiling-induced annual energy losses of about 7% were measured in Santiago, Chile (33°S), a major city with higher rainfall frequency but where urban pollution plays a significant role.

## Introduction

Worldwide, the highest surface irradiance is expected to occur in summer at high altitude sites in the southern hemisphere near the Tropic of Capricorn^1^. Indeed, prior efforts based on available global datasets, a semi-empirical model has pointed the Atacama Desert as the place with the world’s highest solar irradiance^2,3^.

**Figure 1:**
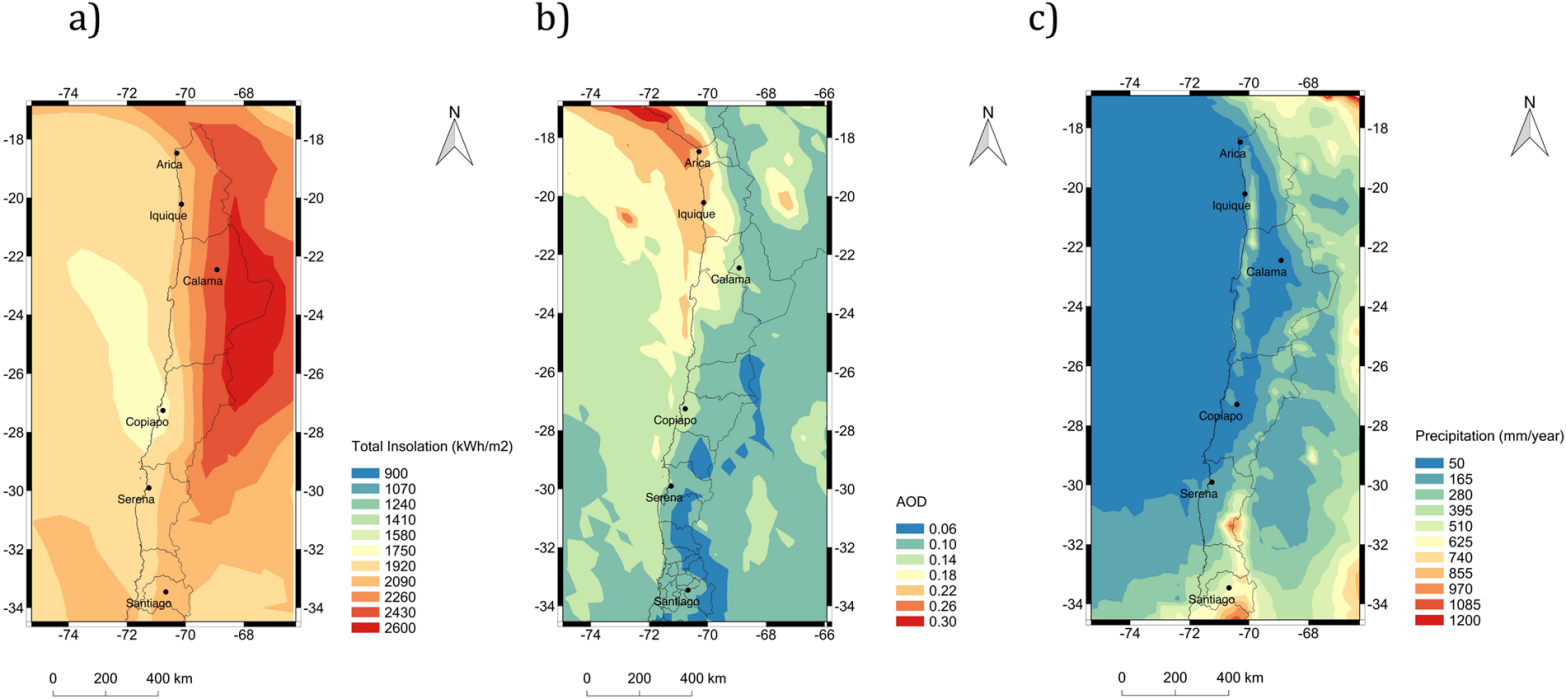
Maps generated by using IDL® (http://www.harrisgeospatial.com/SoftwareTechnology/IDL.aspx#language). (**a**) Annual insolation obtained from the Surface meteorology and Solar Energy (SSE) web portal supported by the NASA Langley Research Center (LaRC) POWER Project. (**b**) Annual average of AOD at 555 nm computed from retrievals of the Multi-angle Imaging SpectroRadiometer (MISR) instrument^39^ (aboard Terra satellite; https:// DOI 10.5067/Terra/MISR/MIL3MAE_L3.004) over the period 2006–2016; (**c**) Average of the annual precipitation over the period 2004–2012 based on the dataset of the Tropical Rainfall Measuring Mission (TRMM) was obtained from the “Observations for Climate Model Intercomparison (obs4MIPs)” project hosted on the Earth System Grid Federation (https://www.earthsystemcog.org/projects/obs4mips/).

The solar potential of the Atacama Desert (see Fig. 1a) has begun to be exploited. Deployment of utility-scale PV power plants soared enormously within recent years; while the PV power capacity was only 3.6 MWp in 2012, it increased to 1.8 GWp by December 2017^4^. Deployment of grid-connected PV systems aimed at self-consumption has been significantly slower, which may be related to some information barriers^5^. Indeed, the cost of operation and maintenance of PV systems aimed at self-consumption in the Atacama Desert is uncertain. In particular, few information on cleaning frequency requirements for counteracting soiling is available for end-users.

Soiling generally arises from the dry deposition of dust and light absorbing particles on the surface of solar energy systems^6–9^. Dry deposition flux depends on dry deposition velocity^9^, which is determined by atmospheric properties as well as the characteristics of the dust and those of the deposition surface^10–12^. There are several models describing the dry deposition velocity of particles as a function of particle size (see a review^10^). Nevertheless, deposition velocity depends on wind speed and the humidity^13^. Higher wind speed increases friction velocity, which accelerates the transport of particulate matter (PM)^10^, while increases in relative humidity lead to hygroscopic growth, which can significantly increase the particle deposition rate^10^.

Dry deposition flux, and in turn the soiling rate, also depends on the PM mass concentration, which has been reported to be well correlated with satellite-derived estimates of the Aerosol Optical Depth (AOD)^14–19^ as well as with ground-based measurements of the AOD^14,20^.

Although few measurements are available for the Atacama desert, aerosols are expected to be abundant in the Atacama Desert due the dry and arid conditions that tend to favor wind-blown dust. However, satellite-estimates of the AOD in the visible range (see Fig. 1b) consistently show relatively low climatological AOD values over the Atacama Desert (ranging from 0.25 in the northern coastal part of the desert to 0.05 over the Andean plateau). Retrievals from the Moderate Resolution Imaging Spectroradiometer (MODIS)^21,22^ allow estimating for the Atacama Desert an annual climatological AOD slightly lower than 0.1 in the visible part of the spectrum. This relatively low AOD roughly agrees with ground-based measurements carried out at the Paranal Observatory (2,635 m altitude, 24°37′S, 70°24′W)^23^. For comparison, the AOD at 500 nm is typically higher than 0.15 at sites in North Africa^24^, while the AOD in the visible range measured at desert sites in northern China ranges from 0.24 to 0.36^25^.

In this paper, we have measured the effects of soiling on the daily energy yield of grid-connected PV modules aimed at self-consumption, deployed in five relatively small cities across a north-south transect of approximately 1300 km in the Atacama Desert: Arica, Iquique, Calama, Copiapo, and La Serena. As shown in Fig. 1c, precipitation in these cities is typically lower than 90 mm per year. For comparison, soiling-induced energy losses were also monitored in Santiago (33°S), a major inland city with higher rainfall frequency and where urban pollution plays a significant role.

Based on the method described by Gostein *et al*.^26^, we measured soiling losses by comparing side-by-side outputs of four co-planar grid-connected crystalline silicon PV modules fitted with micro-inverters. Two of the PV modules of the array were kept clean as a control, while we allowed the other two to naturally accumulate soiling for 12 months.

We found that the combination of high deposition rates and infrequent rainfalls led to annual energy losses that peaked in the northern coastal part of the desert (39% in Arica and 18% in Iquique). In contrast, annual energy losses of 3% or less were measured at relatively high-altitude sites (Calama) and also at locations in the southern part of the desert (Copiapo and La Serena). These figures are consistent with the regional distribution of satellite-derived estimates of the AOD (see Fig. 1b) and with the only prior estimation of the soiling effect at a location in the Atacama Desert (carried out nearby the coastal city of Antofagasta)^27^.

For comparison, annual energy losses due to soiling measured elsewhere are typically in the range of 1–7%^26,28–35^. However, in the case of northern Africa and the Middle East^36–38^, reported energy losses due to soiling are comparable to those measured in the northern coastal part of the Atacama Desert.

Frequent cleaning may be necessary in Arica and Iquique, while in Calama, Copiapo and La Serena, the measured annual energy losses (3% or less) may make the need of cleaning the PV modules more than twice a year unlikely (or economically inconvenient). Methodological details are provided below.

## Materials and Methods

Soiling losses were assessed by comparing side-by-side outputs of four co-planar grid-connected crystalline silicon PV modules (Hareon Solar Technology Co. Ltd. HR-265-18/Cbb 3BB). Each module was fitted with a micro-inverter (Sunny Boy 240-10) that allowed power output to be registered every 15 min. At each location, the tilt angle of the modules was selected according to the latitude. Two of the PV modules of the array were kept clean (through manual washing) as a control, while we allowed the other two to naturally accumulate soiling for 12 months. A similar approach to measure the soiling implies comparing the short-circuit currents (that can be considered a proxy for the effective irradiance received by the modules), or comparing the maximum power production of the modules^26^. However, in most of these cases, prior comparisons involved only two modules (we used four).

**Figure 2:**
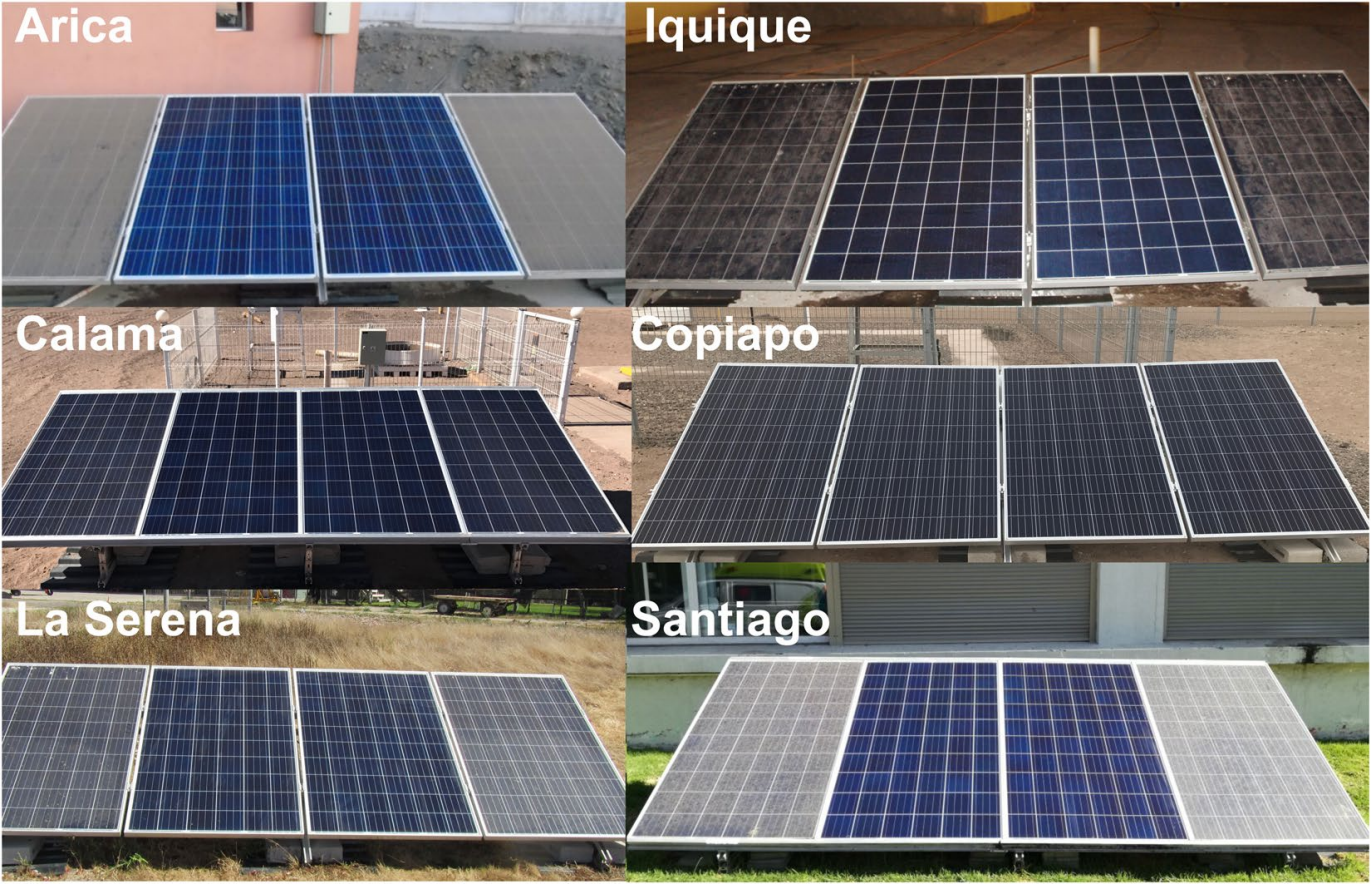
Photographs of the soiling measuring systems. At each location, the central modules of the arrays were manually cleaned while we allowed the other two to naturally accumulate soiling. Please note that these pictures were not taken at the same time and they are not meant to show the typical soiling, since the latter significantly changes during the year. Photographs were taken by the authors (R.R.C., J.J., E.S., D.S., and S.M.).

Figure 2 shows pictures of soiling measuring systems at each location considered in this study: Arica, Calama, Copiapo, La Serena and Santiago. The systems were deployed at the airports/airfields in the case of Calama, Copiapo and La Serena, while they were deployed on university campuses in the case of Arica, Iquique and Santiago. The locations were carefully selected for avoiding any shadow. No significant agricultural activities exist in the Atacama Desert and the systems were deployed relatively far from any major industry. Moreover, although the selected locations can be considered to be urban, soiling is expected to be driven by the desert dust rather than by urban pollution (with the exception of Santiago; see details in Table 1).

**Table 1:** Locations where the soiling effect was measured.

	Lon	Lat	Population	Altitude (m)	Annual Precipitation (mm)	Annual Insolation (kWh/m^2^)
Arica	−70.31	−18.47	221264	20	0.6	2096
Iquique	−70.14	−20.24	191468	50	0.1	1904
Calama	−68.9	−22.49	165731	2293	3.6	2252
Copiapo	−70.78	−27.26	153937	204	13.9	1658
La Serena	−71.2	−29.92	221364	142	74.9	1824
Santiago	−70.68	−33.45	5250565	527	330.1	1886

Data sources as indicated in the section “materials and methods”.

**Figure 3:**
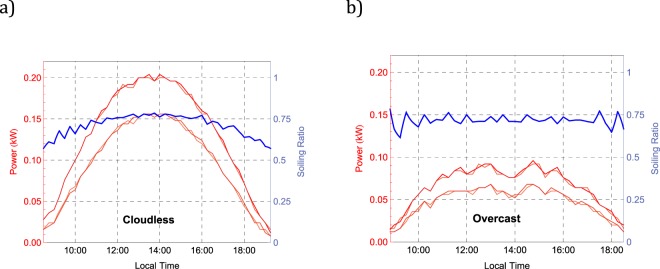
The red curves show the power output of the four PV modules (two clean and two soiled) of our system in Iquique. The blue curve shows the Soiling Ratio (SR) taken in this case as being equal to the ratio between the power output of the soiled modules and the power output of the clean control modules. (**a**) Measurements on January 3rd, 2018 (clear sky conditions). (**b**) Measurements on November 6th, 2017 (overcast conditions).

The red curves in Fig. 3 show the power output of the four PV modules (two clean and two soiled) throughout the day for the array deployed in Iquique. Figure 3a shows measurements from 3 January 2018 (clear sky conditions), while Fig. 3b shows measurements from 6 November 2017 (overcast conditions). Differences in the power output between the soiled modules and the clean control modules are apparent in both plots and can be used to calculate the Soiling Ratio (SR).

In the cases shown in Fig. 3, we took the SR as being equal to the ratio between the power output of the soiled modules and the power output of the clean control modules. The blue curves in Fig. 3a,b show the progress of the SR through the day. It can be observed in Fig. 3a that, early in the morning and in the late afternoon, SR values were slightly lower than at noon, which means that the response of the soiled PV modules changes with the solar zenith angle (SZA). This changes in the angular response are likely due to changes in the optical path length (OPL) of the direct radiation through absorbers and scatterers in the dust layer over the PV module; a shorter OPL (at noon for example) leads to less attenuation in the direct irradiance that reaches the silicon, while a longer OPL leads to more attenuation (which in turn enhances soiling effects). The response of the soiled modules to the nearly isotropic diffuse radiation does not appear to be SZA-dependent such that, when the direct radiation is blocked under cloudy conditions, changes in the SR values throughout the day are less significant (see Fig. 3b).

It can be also observed in both plots in Fig. 3 that SR values (see the blue curves) were noisier early in the morning and in the late afternoon. Noise in our system arises from the nearly random changes (without physical meaning) of up to about ±4 W, in the instant power output rendered by each micro-inverter. This noise complicates the SR assessment by using the power output when the signal is relatively low (early in the morning and late afternoon) but its effect is less significant when comparing the daily total *energy* fed into the grid by the modules.

Indeed, although we registered the power output of the modules every 15 min, in what follows, the SR was computed by comparing the daily energy fed into the grid by the soiled modules with the daily energy fed into the grid by the clean control modules. Including four modules (instead of two) and comparing the daily energy yield (instead of the instant power output) helped to improve the signal to noise ratio (SNR) of our system such that we were able to detect *daily* changes in the SR of about 0.25% (under the typical high irradiance conditions of the Atacama Desert). Using the daily energy yield for computing the SR also allowed us to avoid dealing with the changes in the angular response of the PV modules depending on the cloud conditions (see blue curve in Fig. 3a).

Note that although the modules/inverters were new and expected to be identical, we deployed at each location systems whose *daily* energy yield agreed within a ±0.03 kWh margin (which was tested in the field prior to the experiment). Manual cleaning was carried out at each location at a regular basis (always after 18:00 h LT). In the case of Calama, Copiapo and La Serena, the systems were deployed at airports/airfields such that the cleaning was carried out by the personnel assigned to the airports/airfields. In the case of Arica, Iquique and Santiago, the systems were deployed on university campuses such that university personnel, who were instructed for the task, carried out the cleaning. A team regularly inspected the systems for ensuring that the cleaning was reasonably uniform. Although, we did detect some flaws in the cleaning mostly due to the high personal rotation (especially in that case of Santiago), we were unable to detect any significant affect of these minor flaws in our results.

The soiling led to differences not only in the power output but also in the temperature between the clean and the soiled modules. Although in prior efforts^26^, authors compared temperature-corrected outputs of modules, we chose to apply no correction such that our results for SR accounted for both direct effects (radiation attenuation) and indirect effects (changes in the module’s temperature) of the soiling.

Daily Soiling Loss (SL) was taken as 1 - SR, while the monthly and annual soiling losses were computed by subtracting the monthly and annual energy fed into the grid by clean and soiled modules. Moreover, the daily deposition rate (in what follows also referred to as daily soiling rate) was taken as being equal to the ratio between consecutive daily SR values.

Estimates of the AOD were retrieved from the Multi-angle Imaging SpectroRadiometer (MISR) (aboard Terra satellite). We used the MIL3DAE daily level 3 product from MISR^39^. In the particular case of Santiago and Arica (the only Chilean locations where available), ground-based measurements of the AOD were obtained from the Aerosol Robotic Network (AERONET)^40^.

Precipitation data were obtained over the period 2004–2012 from the dataset of the Tropical Rainfall Measuring Mission (TRMM) of the Observations for Climate Model Intercomparison (obs4MIPs) project^41^. The global insolation data were obtained from the Surface meteorology and Solar Energy (SSE) web portal supported by the NASA Langley Research Center (LaRC) POWER Project^42^. Population and end-user electricity cost at each location was obtained from INE^43^.

## Results

**Figure 4:**
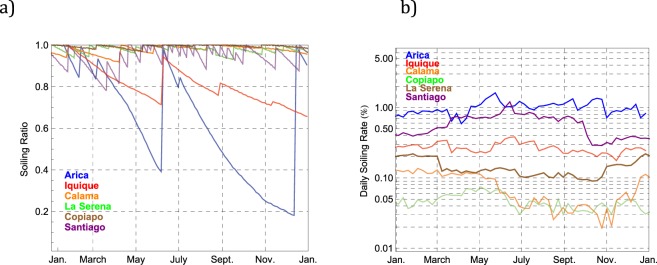
(**a**) Soiling Ratio measurements over a 12-month period in Arica, Iquique, Calama, Copiapo, La Serena and Santiago; (**b**) Daily Soiling Rate computed from the data shown in (**a**).

Figure 4a shows the SR progression through the year in Arica, Iquique, Calama, Copiapo, La Serena and Santiago. At the beginning of the year, the four modules of our soiling measuring system were clean such that the SR was equal to 1 (i.e. the daily energy yields of the soiled and clean modules were the same). The lines stand for the three-day moving average of the daily soiling ratio although, due to its effect on the SR value, the rainy days where excluded from the moving average. Indeed, the effect of natural cleaning by precipitation at each location is apparent in Fig. 4a; by cleaning the soiled modules, precipitation generally resets the systems leading to SR values close to 1. In the time interval between consecutive precipitations, SR (SL) steadily decreased (increased).

Figure 4b shows the progression of the daily deposition rate (or daily soiling rate), which was taken as being equal to the ratio between the consecutive daily SR values shown in Fig. 4a. The lines stand for the five-day moving average of the daily soiling ratio. It can be observed in Fig. 4b that relatively high soiling rates were measured in Arica and in Santiago, while Calama and Copiapo exhibited relatively low soiling rates.

Although the daily soiling rate changed during the year (for example in Arica, it ranged from 0.6 by mid April to 1.4 in late May), the changes in seasonal averages were more significant in Santiago (where the average of the daily soiling rate in autumn-winter doubled with respect to the average in spring-summer) and in Calama (where the average of the daily soiling rate was approximately three times greater in spring-summer than in autumn-winter).

Table 2 shows the seasonal average of the daily soiling rates in Arica, Iquique, Calama, Copiapo, La Serena and Santiago, and the annual energy losses due to soiling at the same locations. As indicated above, annual losses due to soiling were computed by differencing the annual energy fed into the grid by the clean modules and the soiled modules.

**Table 2:** Seasonal averages of the daily soiling rate measured in Arica, Iquique, Calama, Copiapo, La Serena and Santiago; number of self-cleaning events registered in 2017; and annual losses due to soiling at the same locations.

	Daily Soiling Rate (%)						
	Spring-summer	Autumn-winter	Number of Self-Cleaning Events	Annual Energy Loss (%)	Annual Energy Loss (kWh/kWp)	Energy Cost (US$/kWh)	Annual Monetary Loss (US$/kWp)
Arica	**0.75**	**1.12**	3	39	583	0.12	70
Iquique	**0.28**	**0.32**	3	18	271	0.11	30
Calama	**0.11**	**0.03**	5	3	69	0.11	8
Copiapo	**0.05**	**0.03**	10	1	18	0.14	3
La Serena	**0.13**	**0.18**	15	3	51	0.16	8
Santiago	**0.38**	**0.77**	23	7	94	0.19	18

Due to the impact of natural cleaning by rain on annual energy losses, the number of self-cleaning events at each location is also shown in Table 2. Note that events in Table 2 include rainfalls, drizzle and self-cleaning from condensed dew that led to detectable changes in the SR progression. Finally, Table 2 shows the annual monetary loss due to soiling. The latter was estimated from annual energy losses and end-user electricity costs at each location.

## Discussion

### Wind Influence

A strong positive correlation between dry deposition velocity and wind speed has been found in previous studies, for example, in Guangzhou, China^44^. The influence of wind speed on the dry deposition velocity may in turn explain the positive correlation between the dry deposition flux and the measured wind speed in Riyadh City, Saudi Arabia^45^.

**Figure 5:**
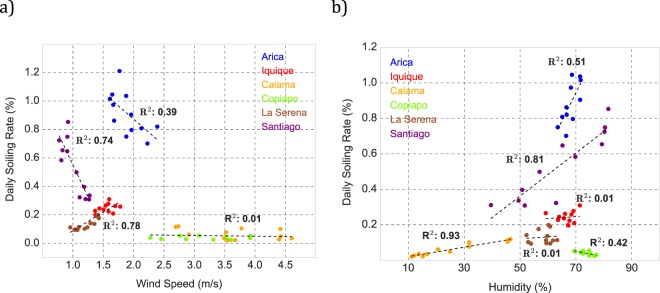
Scatter plot of monthly averages of the daily soiling rate measured in Arica, Iquique, Calama, Copiapo, La Serena and Santiago, and the corresponding: (**a**) monthly wind speed averages; (**b**) monthly relative humidity averages.

Figure 5a shows the scatter plot of the monthly averages of the daily deposition rate (i.e. the daily soiling rate) measured in Arica, Iquique, Calama, Copiapo, La Serena and Santiago, and the corresponding monthly averages of the wind speed. The latter was measured by weather stations set up next to our systems in the case of Santiago, Calama, Copiapo and La Serena, but a couple of km away in the case of Arica and Iquique.

It can be observed in Fig. 5a that the daily soiling rate increased with the wind speed in the case of La Serena and Iquique; the annual averages of the soiling rate were comparable at these locations (0.16%/day in La Serena and 0.31%/day in Iquique). At locations where the annual average of the soiling rate was lower than 0.1%/day (Calama and Copiapo), no significant changes in the deposition rate with the wind speed were detected. It is worth noting that Calama and Copiapo are also the locations (among those considered in this study) with stronger winds and with the greater daily and seasonal changes in direction and speed (monthly wind speed averages range from 2.2 to 3.9 m/s in the case of Copiapo and from 2.6 to 4.6 m/s in the case of Calama). It is remarkable that these changes in winds did not lead to significant changes in the soiling rate.

At locations with an annual average of the soiling rate higher than 0.6%/day (Santiago and Arica; see Fig. 4b and Table 2), the daily soiling rate increased as the wind speed decreased. At least in the case of Santiago, this was expected since in autumn and winter, the sharp reduction in the wind speed complicates ventilation, which in turn leads to a severe increment in PM mass concentration (mainly generated by urban pollution)^15^. This means that, although lowered wind speeds likely aid to lower the dry deposition velocity in autumn and winter, significant increments in the PM mass concentration negate such changes, thereby contributing to increases in the soiling deposition rate.

### Humidity Influence

Relative humidity also influences dry deposition velocity and in turn the soiling rate. Increases in relative humidity lead to hygroscopic growth (increases in particle size), which can significantly increase the particle deposition rate^10^.

Figure 5b shows the scatter plot of the monthly averages of the daily deposition rate (i.e. the daily soiling rate) measured in Arica, Iquique, Calama, Copiapo, La Serena and Santiago, and the corresponding monthly averages of the relative humidity. As in the case of wind speed (see Fig. 5a), relative humidity was measured by weather stations set up next to our soiling measuring systems in the case of Santiago, Calama, Copiapo and La Serena, but several km away in the case of Arica and Iquique.

It can be observed in Fig. 5b that the daily soiling rate increased with relative humidity in the case of Santiago and Arica; the annual averages of the soiling rate were comparable at these locations (0.6%/day in Santiago and 0.9%/day in Arica). A strong positive correlation was also found in the case of Calama (where the soiling rate was lower than 0.1%/day). Note that, at least in the case of Santiago and Calama, these results were expected since at these locations the relative humidity exhibits strong seasonal changes; monthly averages of humidity range from 40 to 80% in the case of Santiago and from 10 to 45% in the case of Calama.

At other locations where seasonal changes in the relative humidity tend to be less significant (Copiapó, La Serena and Iquique), the correlations in Fig. 5b were significantly weaker. Indeed, in the case of coastal locations like La Serena and Iquique, no significant changes in the deposition rate with relative humidity were detected (the annual average of the soiling rate was 0.16%/day in La Serena and 0.31%/day in Iquique).

It is worth noting that at the locations considered in this study we did not detect the cementation process described elsewhere^9^. This process occurs in many regions of the world having both high dust and humidity levels (that may lead to heavy morning dews). At high humidity, water-soluble dust particles on the surface form microscopic droplets of salt solutions that also retain any insoluble particles. When dried by subsequent evaporation, the precipitated salt acts as a cement to anchor insoluble particles to the surface forming a solid, packed cement-like composite^9^. We argue that the low levels of both humidity and aerosols (see next section) do not favor the cementation process in the Atacama Desert.

### Aerosol Influence

Although dry deposition flux is affected by wind speed and humidity (see prior sections), it also depends on particle concentration^10^. Therefore, the daily deposition rate (i.e. the daily soiling rate) and the PM mass concentration are expected to be correlated.

In the case of Santiago, the Ministry of the Environment in Chile maintains an Ambient Air Monitoring Network, but these concentration measurements are not available at other locations considered in this study. However, PM mass concentration has been reported to be well correlated with satellite-derived estimates of the AOD^14,16–19^ and also with ground-based measurements of the AOD^20,46^.

The correlation between PM mass concentration measurements and AOD retrievals has also been previously confirmed in Santiago^15^. Therefore, we tested the correlation between our daily soiling rate measurements and AOD estimates retrieved from satellites and ground-based measurements. Ground-based measurements of the AOD by AERONET-affiliated sunphotometers are available for Arica and Santiago. AOD measurements at 500 nm range from 0.15 to 0.2 in the case of Arica and from 0.1 to 0.2 in the case of Santiago.

**Figure 6:**
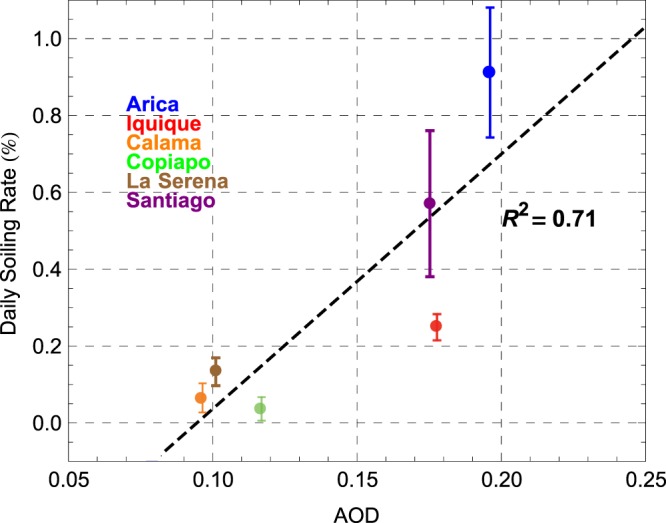
Scatter plot of the annual averages of the daily soiling rate measured in Arica, Iquique, Calama, Copiapo, La Serena and Santiago, and the corresponding annual averages of the AOD estimates. Uncertainty bounds based on the standard deviation of the daily soiling rate measured at each location.

Figure 6 shows the scatter plot of the annual averages of the daily soiling rate measured in Arica, Iquique, Calama, Copiapo, La Serena and Santiago, and the corresponding annual averages of the AOD retrievals. In the case of Iquique, Calama, Copiapo and La Serena, we used MISR-derived estimates at 555 nm, while in the case of Arica and Santiago, we used ground-based AOD measurements at 500 nm carried out by AERONET-affiliated sunphotometers. Dashed line indicates the linear regression. A significant, positive correlation was found.

Figure 6 allows us to address some of the differences in the soiling rate reported in Table 2. For example, although there are some similarities (precipitations and latitude) between Calama and Arica/Iquique, the AOD in Calama is significantly lower than those observed in Arica/Iquique. As shown in Fig. 6, AOD estimates in Calama are rather closer to Copiapo/La Serena, which in turn explains its relatively low soiling rate (see Table 2).

**Figure 7:**
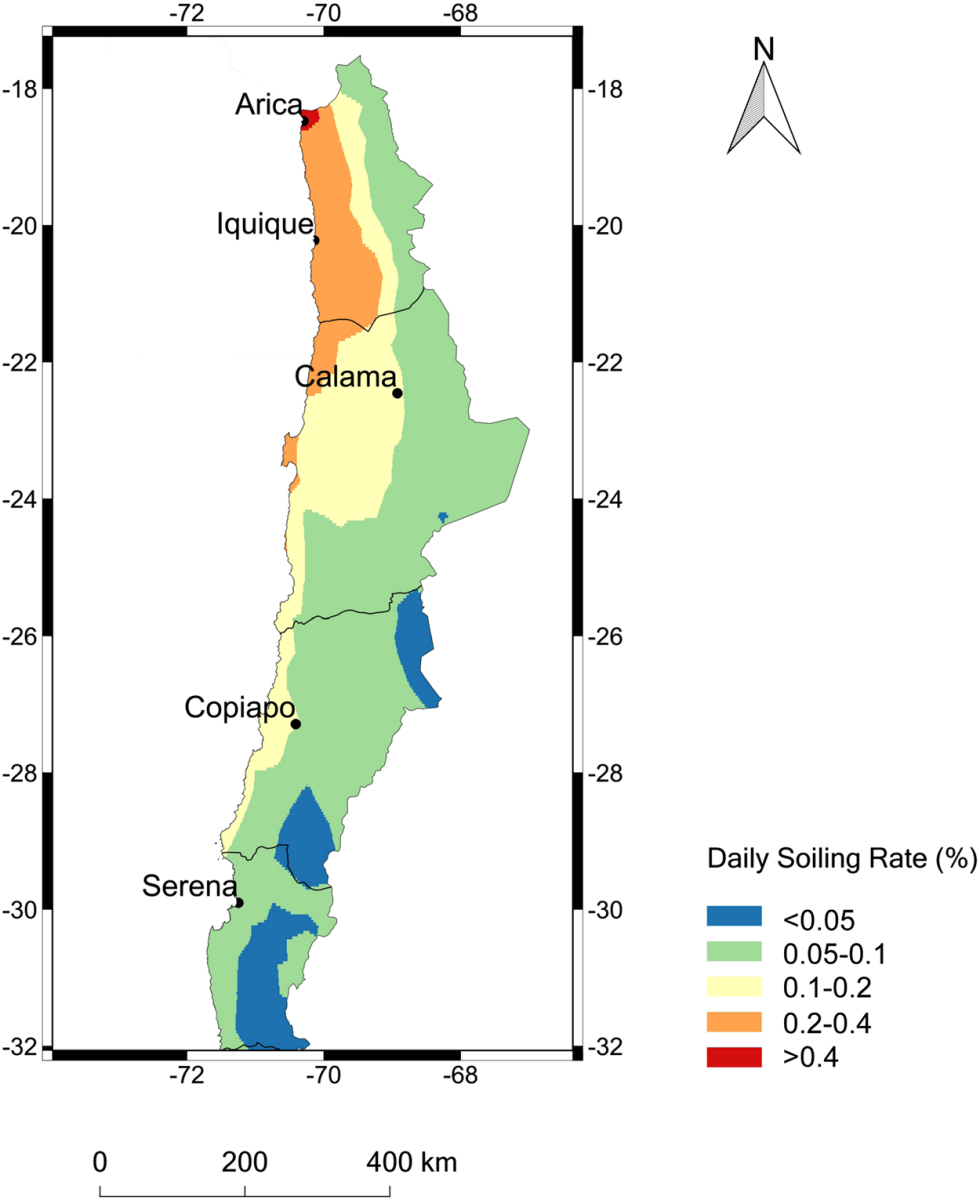
Map generated by using IDL® (http://www.harrisgeospatial.com/SoftwareTechnology/IDL.aspx#language). Daily soiling rate computed from the MISR-derived estimates of the AOD by using the linear regression shown in Fig. 6.

We used the linear regression shown in Fig. 6 in order to estimate the daily soiling rate over the Atacama Desert from the MISR-derived estimates of the AOD. As shown in Fig. 7, the daily soiling rate is expected to be higher than 0.8%/day in the northern coastal part of the desert while it is expected to be lower than 0.3%/day at relatively high-altitude locations in the southern part of the desert. The estimations of the soiling rate shown in Fig. 7 are subjected to significant uncertainties because they are based on measurements conducted only at six locations, and on satellite-derived estimates of the AOD whose accuracy needs to be confirmed (by setting up a sun photomer network aimed at satellite validation).

Indeed, estimates of the AOD retrieved from different satellite instruments show significant differences^47^. In the case of the Atacama Desert, we found significant differences comparing the annual average of AOD values at 555 nm computed from retrievals of the MISR instrument over the period 2006–2016, with the annual average of AOD values at 550 nm derived from retrievals of the MODIS instrument onboard the satellite Terra over the period 2006–2017, especially in the coastal northern part of the Atacama Desert and the eastern slope of the Andes. These differences underlie the need for additional ground-based AOD measurements aimed at validating satellite estimates in the Atacama Desert.

As show in Fig. 6, we found an acceptable correlation (R2 = 0.71) between annual averages of the soiling rate and the corresponding annual averages of the AOD estimates from the MISR instrument. However, we found a weaker correlation when testing the correlation between our soiling rate measurements and AOD retrievals from different satellite instruments (such as the MODIS instrument; we used the Level-3 MODIS products^48^). The same was true when comparing daily and monthly values (instead of annual averages) or when analyzing data of each location separately. There are several factors (including the dry deposition velocity) that can explain the weaker correlation between soiling rate and AOD estimate at monthly and daily time scales. However, as indicated above, the main drawback is likely due to the significant uncertainties associated with satellite–derived AOD estimates over the Atacama Desert.

### Tilt Angle Effect

Attribution efforts aimed at assessing the role of the tilt angle on the soiling rate imply comparing co-located (side-by-side) systems with PV modules at different tilt angles. Although those experiments were beyond the activities considered in our study, based on relevant prior efforts^49,50^, there are several points on this regard that we would like to highlight.

Since in our case the tilt angle of the modules was selected according to the latitude at each location, we expect that the difference in the soiling rate observed between Arica and Santiago should be partially due to the lower tilt angle of the PV modules in Arica (more than 10° lower than in Santiago). Indeed, prior efforts^49,50^ have shown that the transmittance decrease induced on PV modules by similar dust deposition is approximately 10% lower in the case of a tilt angle of 33° (like in Santiago) than in the case of a tilt angle of 18° (like in Arica).

Although the tilt angle effect is indeed significant, it cannot explain the differences in the soiling rate between Arica and Santiago (note that the soiling rate measured in spring-summer was twice in Arica than in Santiago; see Table 2). Moreover, it is unlikely that a slight difference in the tilt angle (of less than 5°) can explain the differences in the soiling rate found between Calama and Iquique (see Table 2). We argue that, although the tilt angle of the modules affects the soiling rate, the high soiling rate observed at the northern most locations (Arica and Iquique) is mainly due to the high aerosol loading at those sites.

### Precipitation Effect

Figure 4b shows that the daily soiling rate in Santiago was comparable with the daily soiling rate in Arica. However, Fig. 4a shows that the soiling ratio progressed differently in these cities such that the annual energy loss was about six times greater in Arica than in Santiago (see Table 2). Most of the differences in the SR progression between Arica and Santiago arise from the different precipitation frequency.

In Arica, precipitation events reset the system three times (in February, March and June). A minor drizzle in early July changed the SR but it was insufficient for resetting the system. The lack of precipitation partially explains why the daily SL in Arica peaked at more than 80% by early December. By mid December the soiling losses in Arica were so significant that they led to a low SNR such that we manually cleaned the modules resetting the system on 15 December. This intervention was not needed at other locations since, as shown in Fig. 4a, rainfall often cleaned the modules and, as expected, the frequency of the cleaning events increased southward.

In Santiago, the daily soiling losses peaked at values close to 20% at the end of the austral summer, but it did not exceed 10% in winter and spring due to 23 rainfalls that often reset the system. For comparison, urban pollution in Mexico City can lead to power losses of up to 15% after two months but, due to subsequent natural cleaning by rainfalls, annual energy loss is Mexico City is estimated to be 3.6%^31^, about half of the annual energy loss measured in Santiago (see Table 2).

**Figure 8:**
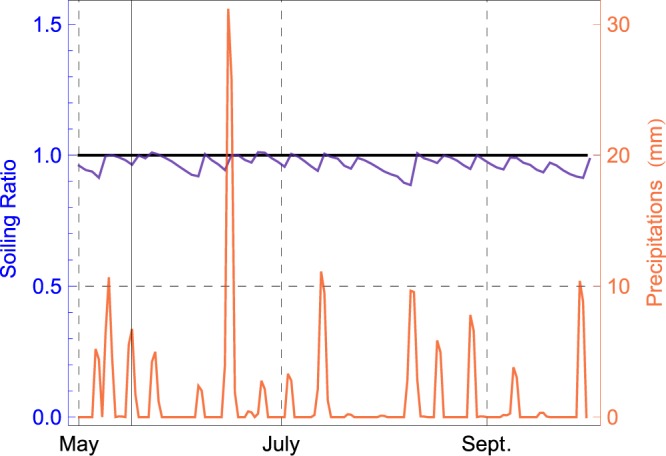
Soiling ratio and precipitations in Santiago from May to October 2017.

Figure 8 shows the progression of both the daily soiling ratio (see blue curve) and the daily precipitation in mm (see red curve) in Santiago from May to October. Comparing both curves allowed us to estimate the rainfall amount required for cleaning the soiled modules (in what follows referred to as rainfall threshold).

Site-specific rainfall thresholds may depend on the type and amount of soiling. However, we found that for SR values higher than 0.9, a precipitation of approximately 0.5 mm was enough for resetting the systems in Arica, Iquique, Calama, Copiapo, La Serena and Santiago.

Since low SR values are associated with locations characterized by a extremely low precipitation frequency (Arica and Iquique), assessing site-specific precipitation thresholds for SR values lower than 0.9 may require years of observation. However, we expect the precipitation threshold for lower values of SR to be in the order of millimeters for the locations considered in this study. For comparison, 4–5 mm of rainfalls are needed to clean solar-tracking PV modules in Navarra, Spain^51^.

The effect of rainfall frequency on the soiling-induced losses can be assessed by comparing the actual annual energy losses (see Table 2) with the potential energy losses due to the soiling (i.e. the losses assuming no precipitation). The latter was calculated by integrating the energy yield through the year of the clean control modules weighted by the daily SR values that were in turn computed (assuming no self-cleaning events) from the measured daily soiling rate shown in Fig. 4b.

**Figure 9:**
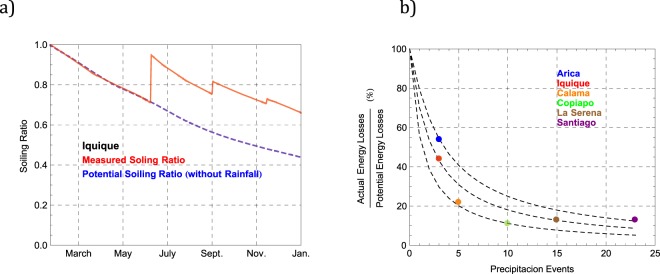
(**a**) Soiling ratio (SR) measured in Iquique (red curve) and SR computed assuming no self-cleaning events (dashed blue curve); (**b**) Fraction of the potential energy losses due to soiling that was actually lost during a 12-month period in Arica, Iquique, Calama, Copiapo, La Serena and Santiago.

For example, Fig. 9a shows the SR values measured in Iquique (see red curve) and the corresponding SR values computed assuming no self-cleaning events (see dashed blue curve). It can be observed in Fig. 9a that without the three self-cleaning events registered in Iquique through the year, the SR would have peaked at about 0.45 at the end of the year (instead of 0.65). By using the output of the clean control modules and the SR values computed assuming no precipitation, we estimated that the annual energy losses would have been 41% (instead of 18% as indicated in Table 2). This means that the three self-cleaning events in Iquique avoided more than half (56%) of the potential energy losses due to the soiling and that the fraction of the potential energy losses due to soiling that was actually lost during a 12-month period in Iquique was only about 44% (100 × 18/41).

Figure 9b shows the results of similar calculations for the other locations considered in this study. The dashed curves link locations with comparable soiling rates. For example, although Santiago and Arica have comparable soiling rates (see Fig. 4b and Table 2), the 23 precipitation events in Santiago avoided 87% of the potential energy loss due to the soiling such that the fraction of the potential energy losses due to soiling that was actually lost during a 12-month period in Santiago was only approximately 13% (see Fig. 9b). In contrast, due to the infrequent precipitation events, the fraction of the potential energy losses due to soiling that was actually lost during a 12-month period in Arica was approximately 54%. The data shown in Fig. 9b enable us to conclude that regardless of the soiling rate, four precipitation events are likely to be enough to avoid 50% or more of the potential annual energy loss due to soiling.

### Cleaning Frequency

Cleaning frequency, either by precipitation or washing, determines the annual energy losses of PV modules due to soiling. Therefore, it is worth paying attention to the optimal cleaning frequency, which will depend on the local soiling rate but also on the cost of cleaning the modules.

**Figure 10:**
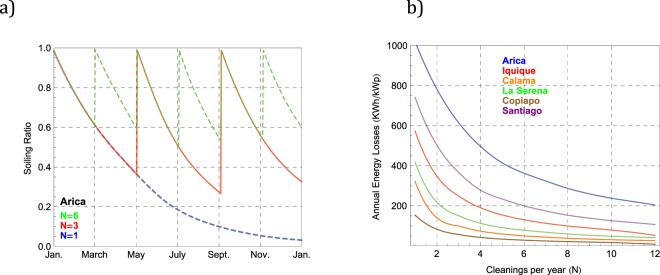
(**a**) Soiling ratio (SR) computed using the daily soiling rate measured in Arica. Each curve stands for the expected SR progression under different cleanings per year (N): annual cleaning (N = 1, dashed blue curve), cleanings at a regular four-month period (N = 3, red curve), and cleanings every two months (N = 6, dashed green curve); (**b**) Energy losses expected under different cleaning frequencies at the locations considered in this study.

Figure 10a shows the expected SR progression through the year, computed by using the daily soiling rate measured in Arica assuming different cleanings per year (N). The dashed blue curve was computed assuming that the modules in Arica were cleaned only once (N = 1), after 12 months. The red curve was calculated assuming either self or manual cleaning at a regular four-month period (N = 3), while the dashed green curve was computed assuming cleanings every two months (N = 6). In a similar way as in Fig. 9a, the cleaning frequency drives the SR values, whose annual average tends to one as N increases.

Figure 10b shows the energy losses expected under different cleanings frequencies at the locations considered in this study. We computed the energy losses by integrating through the year the energy yield of the clean control modules weighted by using daily SR values (see Fig. 10a) that were in turn calculated from the measured daily soiling rate shown in Fig. 4b, but with different values for N. As expected, Fig. 10b shows that the annual soiling loss diminishes as N increases.

The energy losses in Fig. 10b can be used to calculate the annual monetary losses as a function of N, which basically implies using the end-user electricity cost at each location (see Table 2). The monetary loss can be compared with the cleaning cost in order to estimate the optimal cleaning frequency for minimizing the total annual losses (soiling + cleaning).

**Figure 11:**
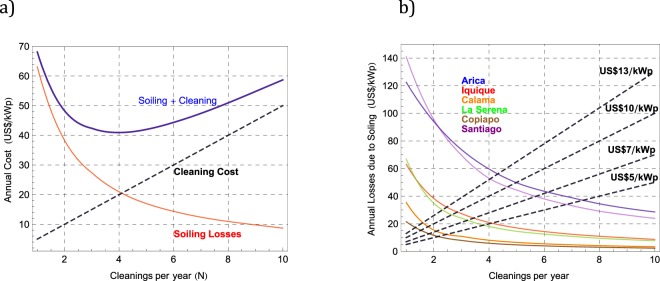
(**a**) Annual soiling losses expected with different cleanings per year (N) in Iquique (red curve); annual costs of cleaning computed under different N values assuming that the cleaning cost is US$5/kWp (dashed black curve); total annual costs under different N values adding up both the soiling losses and the cleaning costs (blue curve); (**b**) Annual soiling losses expected with different cleanings per year (N) at the locations considered in this study. The dashed lines stand for the annual costs associated to N cleanings per year considering different cleaning prices.

For example, the red curve in Fig. 11a stands for the annual monetary losses due to soiling under different cleanings per year (N) in Iquique. This curve was computed by using the corresponding energy losses in Fig. 10b and the end-user electricity cost in Iquique (see Table 2). The dashed black curve in Fig. 11a represents the annual costs of cleaning computed under different values for N with a cleaning price of US$5/kWp. Finally, the blue curve in Fig. 11a stands for the total annual costs under different values for N, which was calculated by adding up both the soiling losses and the cleaning costs. The optimal cleaning frequency in this case is indicated in Fig. 11a by the interception of the curves standing for the soiling losses and the cleaning costs. Indeed, N ~ 4 in Fig. 11a minimizes the total annual costs (soiling + cleaning), which basically means that in Iquique, in the absence of rainfall, solar modules should be cleaned every 3 months (assuming a cleaning price of US$5/kWp).

Figure 11b shows the annual soiling losses expected under different cleanings per year (N) at the locations considered in this study. The dashed lines stand for the annual costs associated to N cleanings per year considering different cleaning prices. The dispersion in the price shown in Fig. 11b (US$5–13/kWp) reflects the actual cleaning prices that end-users in the Atacama Desert will face depending on the particularities and accessibility of their PV system. As in the case of Fig. 11a, the optimal cleaning frequency is indicated in Fig. 11b by the interception of the curves standing for both the soiling losses and the cleaning costs. For example, it can be observed in Fig. 11b that, if the cleaning price is US$7/kWp, the optimal cleaning frequency in Arica is N ~ 6. This means that, in absence of precipitation, solar modules should be cleaned every two months in Arica. In Calama on the other hand, due to low soiling rates in those locations, solar modules only require cleaning approximately every 6 months considering a cleaning price of US$7/kWp.

Figure 11b may allow easily estimating an *optimal* cleaning frequency. However, it should be noted that this estimate does not fully capture the influence of seasonal changes in the soiling rate. Indeed, due to these seasonal changes, the *optimal* cleaning frequency may be different in autumn-winter than in spring-summer. Moreover, although the cleaning frequency retrieved from Fig. 11b may allow planning a cleaning schedule at locations with rare precipitations (which is the case in most of the Atacama Desert), a different approach is required at sites with frequent precipitations.

**Figure 12:**
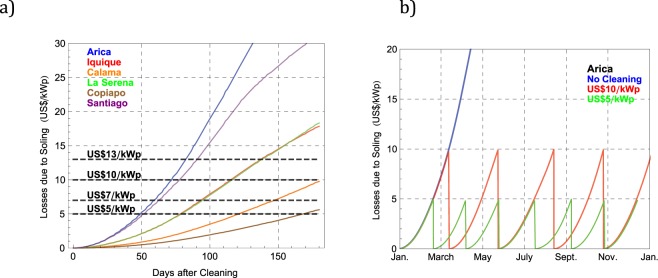
(**a**) Soiling losses progression after cleaning, computed using the daily soiling rate measured in summer at the locations considered in this study. The dashed lines stand for different cleaning prices; (**b**) Expected soiling losses computed by using the daily soiling rate measured in Arica assuming: no cleaning (blue curve), cleanings when losses due to soiling peak at US$10 (red curve), and cleanings when losses due to soiling peak at US$5 (green curve).

Another approach^52–54^ to planning a cleaning schedule is based on cleaning only when the soiling losses are equal to the cost of cleaning. Figure 12a shows the expected progression of the soiling losses, computed by using the daily soiling rate measured *in summer* at the locations considered in this study (see Table 2). The dashed horizontal lines stand for different cleaning costs. This plot allows us to easily retrieve the time required for the soiling losses to equal the cleaning cost, which is indicated by the interception of the curves standing for the soiling losses and the cleaning costs. For example, it can be observed in Fig. 12a that, if the cleaning price is US$7/kWp, the time required in Iquique for the soiling losses to equal the cleaning cost (i.e. the waiting period before next cleaning) is *in summer* approximately 90 days (about 3 months). Indeed, cleaning the modules in Iquique, for example, every month, will not pay off because the increment in energy fed into the grid by frequent washing will not compensate the cleaning costs.

Figure 12b shows the expected progression of the soiling losses during the year, computed by using the daily soiling rate measured in Arica and assuming that cleanings were conducted when the losses due to soiling were equal to an assumed cleaning cost. The red curve was calculated assuming cleanings when losses caused by soiling were equal to US$5/kWp, while the green curve was computed assuming cleanings when soiling losses were equal to US$10kWp. As a reference, the blue curve shows the progression of soiling losses without any cleaning. It can be observed in Fig. 12b that, due to the seasonal changes in the soiling rate measured in Arica, the time required for the soiling losses to equal the cleaning cost (i.e. the “waiting period before next cleaning”) changes during the year. Indeed, due to the seasonal changes in the soiling rate, the “waiting period before next cleaning” will also show seasonal changes.

Table 3 shows the recommended “waiting periods before next cleaning” at the locations considered in this study. These periods are based on the time required for the soiling losses to equal prescribed cleaning costs. We computed the soiling losses according to the measured soiling rates and the end-user electricity costs indicated in Table 2. The waiting periods in Table 3 are shown in months (rather than in days) because of the involved uncertainties (note that our estimations are based on measurements conducted only during one year). It can be also noted in Table 3 that, considering the relatively long “waiting periods”, precipitations will likely allow users to skip some cleanings at locations with relatively frequent self-cleaning events in autumn-winter such as Copiapo, La Serena and Santiago.

**Table 3:** Waiting periods in months before next cleaning according to different cleaning costs.

	US$5/kWp		US$7/kWp		US$10/kWp		US$13/kWp	
	Spring-summer	Autumn-winter	Spring-summer	Autumn-winter	Spring-summer	Autumn-winter	Spring-summer	Autumn-winter
Arica	1.5	1.5	2	2	2	2.5	2.5	3
Iquique	2.5	3	3	4	4	4.5	4.5	5
Calama	4	5	5	6	6	6	>6	>6
Copiapo	5.5	5.5	>6	>6	>6	>6	>6	>6
La Serena	2.5	3	3	4	4	5	5	5.5
Santiago	1.5	2	2	2.5	2.5	3	3	4

### Summary and Conclusions

We have measured the effects of soiling on the daily energy yield of PV modules deployed in five cities across a north-south transect of approximately 1300 km in the Atacama Desert (Arica, Iquique, Calama, Copiapo, and La Serena). For comparison, soiling-induced energy losses were also measured in Santiago (33°S), a major city with higher rainfall frequency and where urban pollution plays a significant role.

Soiling losses were assessed by comparing side-by-side outputs of four co-planar grid-connected crystalline silicon PV modules fitted with micro-inverters. Two of the PV modules of the array were kept clean as a control, while we allowed the other two to naturally accumulate soiling during 12 months.

Relatively high soiling rates (greater than 0.6%/day) were measured in Arica and in Santiago, while Calama and Copiapo exhibited relatively low soiling rates (generally lower than 0.1%/day). The soiling rates measured in La Serena and in Iquique were typically around 0.16%/day and 0.31%/day, respectively. Moreover, the soiling rate changed significantly through the year in Santiago (where the daily soiling rate in autumn-winter doubled with respect to the average in spring-summer) and in Calama (where the soiling rate was nearly three times greater in spring-summer than in autumn-winter).

The annual energy loss due to soiling depends on both the soiling rate and the precipitation frequency. For example, even though the daily soiling rates measured in Santiago and in Arica were comparable, the annual energy loss was about six times greater in Arica than in Santiago (39% versus 7%). This difference largely arises from the 23 rainfalls registered in Santiago in 2017 (versus only three drizzle events detected in Arica in 2017).

Reducing soiling losses may require washing of PV modules but at locations such as Calama, Copiapo and La Serena, the measured annual energy losses (3% or less) may make cleaning the PV modules more than twice a year economically inconvenient. The combination of high soiling rates and infrequent precipitation events will likely make manual cleaning in Arica and Iquique necessary. Although the optimal washing frequency will depend on the monetary cost of cleaning, we estimate that in the absence of precipitation and considering a cleaning price in the range US$5–10/kWp, solar modules should be cleaned every 3–4 months in Iquique and every 45–75 days in Arica.

## Data Availability

The datasets generated and analyzed during the current study are available from the corresponding author on reasonable request.
